# Recurrent Selection with Sub-Lethal Doses of Mesotrione Reduces Sensitivity in *Amaranthus palmeri*

**DOI:** 10.3390/plants10071293

**Published:** 2021-06-25

**Authors:** Jason K. Norsworthy, Vijay K. Varanasi, Muthukumar Bagavathiannan, Chad Brabham

**Affiliations:** 1 Department of Crop, Soil, and Environmental Sciences, University of Arkansas, Fayetteville, AR 72701, USA; vijaya.varanasi@gmail.com (V.K.V.); chad.brabham@fmc.com (C.B.); 2 Department of Soil and Crop Sciences, Texas A & M University, College Station, TX 77843, USA; muthu@tamu.edu

**Keywords:** recurrent selection, weed resistance, non-target-site resistance, sub-lethal herbicide application, HPPD inhibitors, gene expression

## Abstract

*Amaranthus palmeri*, ranked as the most prolific and troublesome weed in North America, has evolved resistance to several herbicide sites of action. Repeated use of any one herbicide, especially at lower than recommended doses, can lead to evolution of weed resistance, and, therefore, a better understanding of the process of resistance evolution is essential for the management of *A. palmeri* and other difficult-to-control weed species. *Amaranthus palmeri* rapidly developed resistance to 4-hydroxyphenylpyruvate dioxygenase (HPPD) inhibitors such as mesotrione. The objective of this study was to test the potential for low-dose applications of mesotrione to select for reduced susceptibility over multiple generations in an *A. palmeri* population collected from an agricultural field in 2001. F_0_ plants from the population were initially treated with sub-lethal mesotrione rates and evaluated for survival three weeks after treatment. All F_0_ plants were controlled at the 1× rate (x = 105 g ai ha^−1^). However, 2.5% of the F_0_ plants survived the 0.5× treatment. The recurrent selection process using plants surviving various mesotrione rates was continued until the F_4_ generation was reached. Based on the GR_50_ values, the sensitivity index was determined to be 1.7 for the F_4_ generation. Compared to F_0_, HPPD gene expression level in the F_3_ population increased. Results indicate that after several rounds of recurrent selection, the successive generations of *A. palmeri* became less responsive to mesotrione, which may explain the reduced sensitivity of this weed to HPPD-inhibiting herbicides. The results have significance in light of the recently released soybean and soon to be released cotton varieties with resistance to HPPD inhibitors.

## 1. Introduction

*Amaranthus palmeri* S. Watson (Palmer amaranth) is a problematic weed in North America, especially in mid-southern United States, due to its rapid growth rate, high fecundity, and a strong tendency to evolve resistance to several herbicide sites of action (SOA) [1,2,3]. *Amaranthus palmeri* has extensive genetic diversity being a dioecious and obligate outcrosser, which favors widespread herbicide resistance evolution in this species [4].

One of the herbicide classes to which *A. palmeri* rapidly evolved resistance was the 4-hydroxyphenylpyruvate dioxygenase (HPPD) inhibitors, commonly known as bleachers. Two of the first HPPD-inhibiting herbicides registered in the USA were isoxaflutole and mesotrione in 1998 and 2001, respectively. Resistance to HPPD inhibitors was first reported in *Amaranthus tuberculatus* (Moq.) Sauer (waterhemp) in 2009, and then in Palmer amaranth in 2012 [5,6]. Herbicides that inhibit HPPD are extensively applied preemergence and postemergence, mainly in maize and grain sorghum, to control a broad range of weed species, including *A. palmeri*. The HPPD-inhibiting herbicides have proven effective for controlling several glyphosate-, acetolactate synthase (ALS)-, and photosystem (PS) II-inhibitor-resistant weed biotypes [7,8]. However, due to the increasing incidents of HPPD-inhibitor resistance, a better understanding of weed resistance evolution to this chemistry is essential for sustaining its utility. This is especially important considering the recent commercialization of soybean with resistance to HPPD-inhibiting herbicides (LL^®^GT27™) as well as the upcoming launch of cotton resistant to HPPD inhibitors (known as HPPDi cotton) [9,10].

4-Hydroxyphenylpyruvate dioxygenase catalyzes the oxidative decarboxylation of 4-hydroxyphenylpyruvate (4-HPP) to form homogentisate (HGA) in the tocopherol biosynthetic pathway. Mesotrione (2-[4-(methylsulfonyl)-2-nitrobenzoyl]-1,3-cyclohexanedione) inhibits the HPPD enzyme, preventing the conversion of tyrosine to plastoquinone (PQ) and α-tocopherol [11]. Plastoquinone is an essential component of carotenoid biosynthesis and the limited or no availability of PQ affects the downstream synthesis of carotenoids, which are essential for two critical roles during photosynthesis: light harvesting and protection against photooxidative damage [12,13]. Mesotrione treatment in sensitive species leads to reduced pigment biosynthesis and impaired chloroplast development.

Recommended or allowed use rates for herbicides are provided on the product labels to ensure they are consistently effective over broad environmental settings [14]. However, under certain conditions, reduced herbicide rates have been known to achieve comparable weed control levels in some cropping systems. From an economic standpoint, this creates a motive for growers with large acreages in developed countries to lower their herbicidal rates for attaining acceptable weed control levels [15]. However, in reality, weed populations are known to display high levels of genetic diversity and phenotypic plasticity, capable of tolerating low herbicide rates and control measures.

There are two major herbicide resistance mechanisms commonly reported in weeds, target- and non-target-site based. In general, spraying herbicides at higher rates favors the development of target-site, whereas lower than recommended rates favor non-target-site metabolic or creeping resistance [16,17]. Resistance to HPPD inhibitors in *A. palmeri* and *A. tuberculatus* was mainly found to be metabolic in nature [18,19].

Herbicides at sub-lethal rates can rapidly lead to non-target-site resistance in cross-pollinated species [15,20]. Use of sub-lethal herbicide rates is known to result in selection and accumulation of non-target-site resistance mechanisms in subsequent generations [20]. Sub-lethal rates accumulate minor resistance conferring alleles (i.e., polygenic resistance), leading to the evolution of resistant phenotypes over several generations [21]. An increase in cases of non-target-site resistance in outcrossing species due to reduced herbicide usage has been documented in recent years [15,20,22,23]. Previously, low-dose recurrent selection leading to herbicide resistance has been reported for glyphosate and dicamba in *A. palmeri*, acetyl CoA carboxylase inhibitors in *Lolium rigidum* Gaudin (rigid ryegrass), and 2,4-D in *Raphanus raphanistrum* L. (wild radish) [15,20,24,25,26]. However, the consequences of low-dose selection for HPPD inhibitors in weed species, especially in *A. palmeri*, are not known. It has been suggested that the tolerance of *A. palmeri* to herbicides may be progressively increasing with each generation when applied at less than recommended rates [24].

Herbicide resistance resulting from a low-dose recurrent selection process is polygenic and metabolic in nature [20,21]. Previous studies with diclofop-methyl in rigid ryegrass have indicated rapid herbicide metabolism in low-dose selected populations (R1, R2) when compared to unselected populations (S1, S2) [27]. More recently, several studies have also associated increased target-site gene expression with the rapid detoxification process. One such study confirmed increased mesotrione metabolism and HPPD transcript levels in *A. palmeri* under high temperature conditions [28]. Increased HPPD gene expression (4- to 12-fold) and rapid mesotrione metabolism in resistant *A. palmeri* populations from Kansas and Nebraska have been reported [19]. However, to date, no one has studied the target-site gene expression in successive generations of low-dose recurrent selection in weed species. Does gene expression correlate with reduced herbicide sensitivity in successive generations?

The objectives of this study were to determine (1) the potential for sub-lethal doses of mesotrione to select for reduced susceptibility over multiple generations in an *A. palmeri* population collected in 2001, and (2) characterize the target-site HPPD gene expression in successive recurrent selections.

## 2. Results

### 2.1. Low-Dose Mesotrione Selection

**Figure 1:**
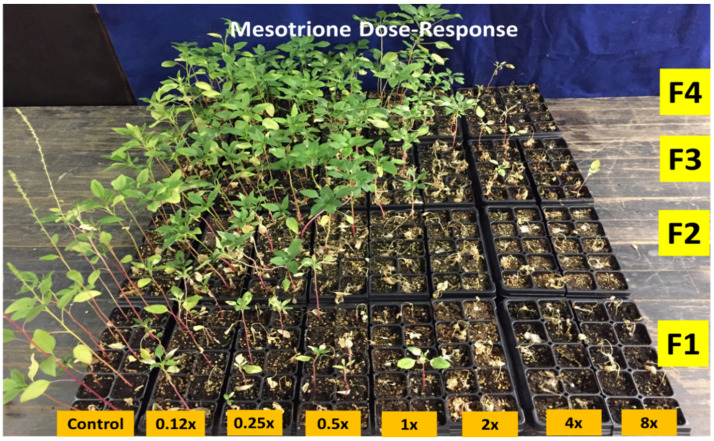
Generations of *A. palmeri* (F_1_, F_2_, F_3_, and F_4_) treated with different mesotrione rates (x = 105 g ai ha^−1^). Picture taken 3 weeks after treatment.

**Figure 2:**
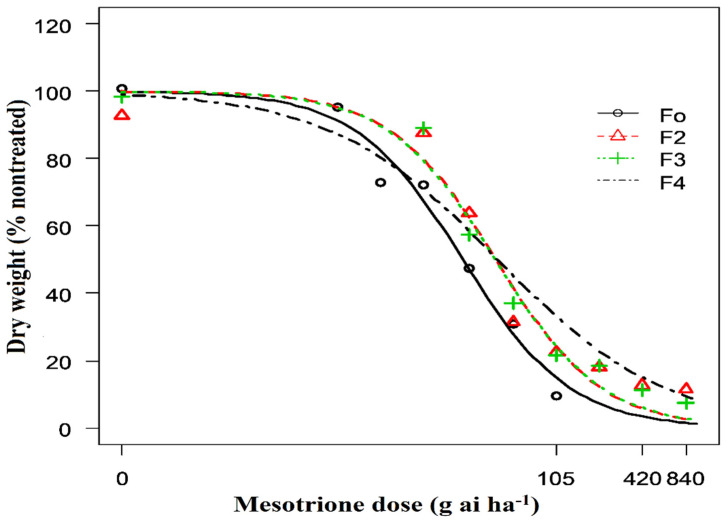
Dose–response curves for generations of *A. palmeri* (F_0_–F_4_) selected following sub-lethal doses of mesotrione in the greenhouse.

Recurrent selection for two generations (F_2_) resulted in an increase in the number of individuals surviving 1.0 (56%) and 1.5× (37%) rates (Table 1). Even at F_3_, there were a significant number of survivors (31 and 10%) at 1.5 and 2.0× rates. These results indicate that after repeated recurrent selections, F_2_′s and F_3_′s became less susceptible to mesotrione compared to the initial F_0_ generation. Dose–response curves and the corresponding GR_50_ values revealed reduced sensitivity to mesotrione for F_4_ when compared to F_0_ (F_4_/F_0_ = 1.7) (Table 2, Figure 1 and Figure 2).

### 2.2. HPPD Gene Expression

**Figure 3:**
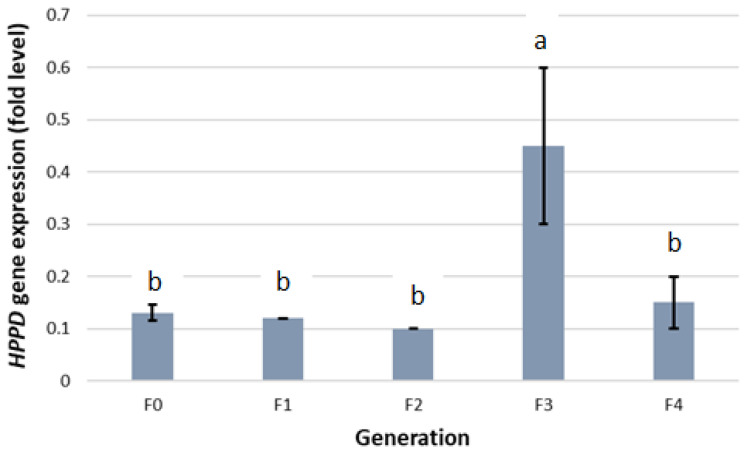
HPPD gene expression in *A. palmeri* generations (F_0_, F_1_, F_2_, F_3_, F_4_). Gene expression was measured relative to the reference gene (*β*-*tubulin*) and expressed in fold levels. Data represent means of biological replicates, and error bars represent SE. Different lowercase letters above bars represent statistical difference in treatments based on Tukeys HSD test.

In this study, increased HPPD expression levels were observed in the F_3_ generation (Figure 3). HPPD expression remained low (near to F_0_ levels) until the F_2_, and a sudden spike was observed at the F_3_ generation. The population had a 1.5-fold reduction in response to mesotrione at F_3_.

## 3. Discussion

The recurrent selections conducted on the 2001 population resulted in a shift in reduced sensitivity to mesotrione (up to 1.7-fold for the F_4_ generation). These data reveal that application of sub-lethal mesotrione rates over subsequent generations leads to reduced *A. palmeri* sensitivity to the herbicide, eventually leading to resistance based on the survival of offspring from selection to a labeled field rate of mesotrione. Factors such as biotype (biological) and herbicide dose (operational) are known to greatly impact the rate of resistance evolution [15].

Herbicide resistance due to sub-lethal selection occurs more rapidly in cross-pollinated than in self-pollinated species [21]. *Amaranthus palmeri,* being an obligate, cross-pollinated species due to dioecy, is more prone to herbicide resistance resulting from sub-lethal selection. The accumulation and increase in the frequency of genes having additive effects leads to a rise in reduced sensitivity to herbicides in cross-pollinated weed populations [15,29]. In general, the frequency of resistance alleles initially present in any population is a key driving force for evolution of herbicide resistance [30]. Sensitivity of the final selection revealed a GR_50_ of 41.22 g ai ha^−1^, which was significantly greater than the initial GR_50_ of 23.57 g ai ha^−1^.

The reduced mesotrione susceptibility of *A. palmeri* observed in this study somewhat matches the reduced susceptibilities observed earlier for dicamba, 2,4-D, and glyphosate in this species following recurrent sub-lethal selection [24,26]. For *Lolium rigidum*, another outcrossing species, three cycles of low-dose selection resulted in a 56-fold increase in diclofop-methyl resistance [20], which may indicate that some major alleles were responsible for resistance. In the case of dicamba, three cycles of repeated selection were necessary to result in individuals capable of surviving the label rate (560 g ae ha^−1^) [26]. However, in our study, individuals surviving mesotrione at 105 g ai ha^−1^, the labeled rate, was reached in the second generation (F_2_) (Table 1). This shows that compared to other herbicide SOA, there is a more rapid evolution of sub-lethal dose-mediated mesotrione resistance in *A. palmeri*. The rapid selection for resistance to HPPD herbicides is not surprising considering the failure of a labeled rate of mesotrione (105 g ai ha^−1^) and tembotrione (92 g ai ha^−1^) to control *A. palmeri* accessions throughout Arkansas in recent screenings [31,32].

An increase in HPPD gene expression, a target-site based mechanism, leads to increased mesotrione resistance following low-dose selection. Nakka et al. [19] have previously reported increased HPPD gene expression in mesotrione-resistant *A. palmeri* populations, while suggesting the role of cytochrome P450 monooxygenases (CYP450s) in the detoxification process. Cytochrome P450s have also been implicated in the non-target-site-based resistance mechanism involving metabolism of mesotrione and other HPPD inhibitors in *A. tuberculatus* [18,33,34]. In addition to the target-site mechanism (HPPD overexpression) observed here, it is also likely that non-target-site (metabolic) resistance mechanisms may be contributing to the resistance in this population, which was not investigated in this study.

An increase in HPPD gene expression does not appear to be the likely cause of resistance in this *A. palmeri* population following recurrent selection. Findings from this research lead to the conclusion that it takes multiple cycles of low-dose recurrent selection for favorable alleles (most likely from the CYP450 family) to come together and have significant impact on mesotrione resistance, at least at the population size evaluated. Future efforts should evaluate expression of CYP450 genes and the effectiveness of the CYP450 inhibitor malathion in reversing resistance to HPPD-inhibiting herbicides. There should also be efforts to quantify mesotrione metabolism on recurrently selected populations to determine if the resistance level observed at F_3_ translates to rapid detoxification when compared to unselected populations (F_0_). If overexpression of CYP450 genes is found and enhanced metabolism is documented, efforts should focus on identifying the specific genes responsible for this non-target-site resistance mechanism. It will be intriguing to see if this mesotrione-resistant population would also be cross-resistant to other herbicide chemistries. Additionally, data for mesotrione resistance in *A. palmeri* using this population could be useful for modeling the evolution of resistance to HPPD-inhibiting herbicides and developing management strategies that mitigate risk for polygenic resistance evolution.

Under greenhouse conditions, the use of sub-lethal mesotrione rates over several generations selected for resistance in this population was demonstrated. Recent screenings for sensitivity of *A. palmeri* accessions in Arkansas to mesotrione and tembotrione revealed that a highly variable response was present among sampled sites [31,32]. The evolution of mesotrione resistance under field conditions is possible if reduced herbicide rates are applied for economic reasons, weeds emerge as soil-applied activity of the herbicide partially dissipates, the herbicide is applied to larger weeds than recommended, or environmental conditions are such that they lead to reduced herbicide efficacy [35,36,37] because all these scenarios may allow for the selection and accumulation of minor effect resistance conferring alleles in the population. One strategy that continues to be recommended is to follow the label in all instances and diversify weed control tactics as much as possible [36]. Using diverse herbicides with residual activity for preemergence instead of relying solely on postemergence weed control integrated with non-chemical measures would also help with long-term resistance management.

## 4. Materials and Methods

### 4.1. Parental Populations (F_0_)

Seeds of *A. palmeri* (F_0_) were collected in Arkansas, United States, in 2001 from an agricultural field site. The field history of this site was unknown, but it is unlikely that these fields had ever been treated with mesotrione or another HPPD-inhibiting herbicide considering the lack of significant maize hectares in Arkansas prior to 2001 and the fact that HPPD herbicides were not widely used in Arkansas maize production prior to this time. When samples were collected, approximately 15–20 female plants were harvested per field, and seeds from these plants were combined into a composite sample, representing the population. Seeds were placed in dry storage at 4 °C.

The susceptibility of the F_0_ population was confirmed in 2016 in a preliminary greenhouse study using mesotrione (Callisto^®^, Syngenta Crop Protection, Greensboro, NC, USA) at 105 g ai ha^−1^ (1× rate). Greenhouse experiments, including selection, dose–response, and gene expression, were conducted during 2017 and 2018 at the Altheimer Laboratory, University of Arkansas, Fayetteville. The seed obtained from each selection cycle was planted in plastic trays (25 cm × 55 cm), and after germination, seedlings at the 2-leaf stage were transplanted into 50-cell plastic trays (tray size: 25 cm × 55 cm) filled with potting mix (Sunshine premix No. 1^®^; Sun Gro Horticulture, Bellevue, WA, USA). A 16 h photoperiod using high-pressure sodium lamps (400 µmol m^−2^ s^−1^), 35/25 °C day/night temperature regime, and a daily watering schedule was maintained throughout the greenhouse study.

### 4.2. Generation of F_1_–F_4_

All selections took place by treating 4- to 6-leaf *A. palmeri* plants grown in a greenhouse. Two rates of mesotrione were chosen for each selection, with the goal of achieving approximately 90–95% mortality across the rates. The F_0_ plants were treated with mesotrione at 0.5 and 1× rates (Table 1). F_0_ survivors from the 0.5× rate were allowed to grow in isolation in the greenhouse and cross-pollinate to produce F_1_ seed. In the next selection cycle, F_1_ plants were treated with 0.75 and 2× doses of mesotrione, and survivors from both doses were allowed to produce F_2_ seed in isolation (Table 1). The F_2_ seed from the 0.75 and 2× doses were combined and used for further selection cycles with increasing mesotrione rates until F_3_ and F_4_ seeds were generated (Table 1). Each selection cycle in the greenhouse was completed in a five- to six-month period: January–May 2017 (F_1_), June–November 2017 (F_2_), December 2017–April 2018 (F_3_), and May–October 2018 (F_4_).

### 4.3. Dose–Response Experiments

Dose–response assays were run on the selections in the greenhouse to determine the level of mesotrione resistance in each cycle (as discussed above). The experiment was a randomized complete block design with 3 replications (10 plants replication^−1^) and was conducted in two runs. Seed obtained from F_0_, F_2_ (0.75× selection), F_3_ (1×), and F_4_ (1.5×) was used in the dose–response assays. Adequate F_1_ seed was not available and therefore was not included in the dose–response experiment. 

*Amaranthus palmeri* seedlings at 4- to 6-leaf growth stage were treated with increasing rates of mesotrione. The following mesotrione rates were used for the F_0_ generation: 0, 0.03, 0.06, 0.12, 0.25, 0.5, and 1.0×. The rates were chosen based on the initial sensitivity of the F_0_ population. For F_2_ through F_4_ generations, mesotrione rates utilized for the dose–response study were: 0, 0.12, 0.25, 0.5, 1.0, 2.0, 4.0, and 8.0×. Crop oil concentrate (Superb^®^ HC, Winfield Solutions, St. Paul, MN, USA) at 1% *v/v* was included as an adjuvant in all treatments as recommended by the product label [29]. A research track sprayer equipped with two 800067 flat-fan spray nozzles (TeeJet spray nozzles; Spraying Systems Co., Wheaton, IL, USA) calibrated to deliver 187 L ha^−1^ of herbicide solution at 269 kPa, moving at a speed of 1.6 km h^−1^, was used for applying treatments to plants. Percent survival (based on regrowth) and aboveground dry biomass data were collected 3 wk after treatment (WAT). Aboveground fresh biomass from the plants was harvested in paper bags, oven-dried at 65 °C for 3 d, and then expressed as a percentage of the nontreated for dose–response curves.

### 4.4. Data Analysis

In this study, there was no significant treatment by run interaction (*p* > 0.05). Therefore, dose-response data were pooled between the two experimental runs for analysis. The dose-response analysis was conducted on the aboveground dry biomass data using the drc package in R 3.1.2 [38]. A four-parameter log-logistic model [39] was used to define the relationship between herbicide rates used and the corresponding dry biomass:

(1) $$Y=C+\frac{D-C}{1+\exp\{b[\log(x)]-\log(GR_{50})\}}$$

where *Y* is the response (aboveground dry biomass) expressed as a percent nontreated, *C* and *D* are the lower and upper asymptotes of *Y*, *b* is the slope of the curve around the GR_50_ (herbicide dose required for 50% reduction in biomass), and *x* is the herbicide dose. Resistance or sensitivity index (resistance/susceptibility (R/S)) for each generation was calculated based on the respective GR_50_, using F_0_ as the base population (Table 2).

### 4.5. RNA Extraction, cDNA Synthesis, and Quantitative PCR

The developing, non-fully expanded youngest leaf was harvested 3 d after treatment (DAT) (mesotrione at 1×) in 1.5 mL microfuge tubes (Thermo Fisher Scientific, Waltham, MA, USA) and stored at −80 °C for RNA extraction. The frozen leaf tissue was homogenized in liquid nitrogen using a prechilled mortar and pestle. The powdered tissue was transferred to a prechilled 1.5 mL microcentrifuge tube, and total RNA was isolated using the recommended protocol of TRIzol^®^ reagent (Thermo Fisher Scientific, Waltham, MA, USA). To remove contamination due to genomic DNA, ribonucleic acid was treated with DNase 1 enzyme (Thermo Fisher Scientific, Waltham, MA, USA). Total RNA was quantified using a spectrophotometer (NanoDrop 1000, Thermo Fisher Scientific, Waltham, MA, USA) and its integrity checked using agarose gel (1%) electrophoresis. About 1 μg of the total RNA was converted to complementary DNA (cDNA) using a RevertAid First Strand cDNA Synthesis Kit (Thermo Fisher Scientific, Waltham, MA, USA).

A SYBR Green quantitative PCR (qPCR) assay was performed to study the expression of the HPPD gene in successive generations (F_0_ to F_3_/F_4_) of the two populations after mesotrione application. The qPCR reaction mix (10 μL) contained 5 μL of PowerUP SYBR Green mastermix (Applied Biosystems, Waltham, MA, USA), 1 μL each of forward and reverse gene specific primers (5 μM), 1 μL of nuclease-free water, and 2 μL of the target cDNA. The forward and the reverse gene specific primers for amplifying the HPPD were 5′-TGAAACCCGAAAATCAACCCG-3′ and 5′-TGTATCTGGAACCTGTTTACG-3′. The primers were designed based on the *A. tuberculatus* sequence information available online (KY689232.1) [40]. *β-tubulin* was used as a reference gene for normalizing the HPPD gene expression [28]. The qPCR was run at 50 °C for 2 min, 95 °C for 2 min, and 40 cycles of 95 °C for 30 s, and 59 °C for 1 min, respectively. The qPCR protocol was followed by a melt curve profile to determine the specificity of the qPCR product. A 2^ΔCt^ method was utilized to measure the relative HPPD: *β-tubulin* expression, where Ct is threshold cycle and ΔCt is Ct_Reference gene (*β-tubulin*)_ − CT_Target gene (*PPX2*)_. Standard errors were generated using three biological and three technical replicates. Change in levels of HPPD gene expression in F_1_, F_2_, F_3_, and F_4_ generations were compared to the base F_0_ levels for analysis. Gene expression data were analyzed by ANOVA and Tukey’s HSD test.

## Figures and Tables

**Table 1:** A 2001 *A. palmeri* population (F_0_) used for generating F_1_, F_2_, F_3_, and F_4_ lines. The table shows the mesotrione rates and the number of seedlings used in successive generations of the recurrent selection process. The survivors from each generation were allowed to cross and produce next generation seed. Dead/alive counts were recorded 3 weeks after treatment.

	Mesotrione Dose (x) (1× = 105 g ai ha^−1^)	# of Seedlings Treated	Survivors (%)
F_0_	0.5	200	2.5
	1.0	200	0
F_1_	0.75	275	2.5
	2.0	275	1.4
F_2_	1.0	250	56
	1.5	200	37
F_3_	1.5	115	31
	2.0	100	10

**Table 2:** The effective mesotrione doses (g ha^−1^) that caused 50% growth inhibition (GR_50_) in different generations of *A. palmeri* collected from an agricultural field.

Generation	GR_50_^a^	Sensitivity Index ^b^
F_0_	23.57 (2.00)	-
F_2_	39.96 (3.33)	1.5 (F_2_/F_0_)
F_3_	39.60 (3.33)	1.5 (F_3_/F_0_)
F_4_	41.22 (5.07)	1.7 (F_4_/F_0_)

^a^ Numbers in parenthesis of GR_50_ values represent SE; ^b^ Sensitivity index for mesotrione calculated based on the GR_50_ values for each generation. F_0_ was used as the base population for the calculation.

## Data Availability

Not applicable.
